# Multiplex PCR performed of bronchoalveolar lavage fluid increases pathogen identification rate in critically ill patients with pneumonia: a pilot study

**DOI:** 10.1186/s13613-014-0035-7

**Published:** 2014-11-25

**Authors:** Jean-Luc Baudel, Jacques Tankovic, Redouane Dahoumane, Fabrice Carrat, Arnaud Galbois, Hafid Ait-Oufella, Georges Offenstadt, Bertrand Guidet, Eric Maury

**Affiliations:** 1 Assistance Publique - Hôpitaux de Paris (AP-HP), Hôpital Saint-Antoine, Service de Réanimation Médicale, Paris 75012, France; 2 AP-HP, Hôpital Saint-Antoine, Service de Microbiologie, Paris 75012, France; 3 Inserm, UMR 707, Paris 75012, France; 4 UPMC - Université Paris 06, Paris 75012, France

**Keywords:** Multiplex PCR, SeptiFast®, Bronchoalveolar lavage, Antibiotic therapy, Prior antibiotic treatment, Community-acquired pneumonia, Hospital-acquired pneumonia, Ventilator-acquired pneumonia

## Abstract

**Background:**

In critically ill patients with pneumonia, accurate microorganism identification allows appropriate antibiotic treatment. In patients undergoing bronchoalveolar lavage (BAL), direct examination of the fluid using Gram staining provides prompt information but pathogen identification accuracy is low. Culture of BAL fluid is actually the reference, but it is not available before 24 to 48 h. In addition, pathogen identification rate observed with direct examination and culture is decreased when antibiotic therapy has been given prior to sampling. We therefore assessed, in critically ill patients with suspected pneumonia, the performance of a multiplex PCR (MPCR) to identify pathogens in BAL fluid. This study is a prospective pilot observation.

**Methods:**

We used a MPCR detecting 20 types of microorganisms. Direct examination, culture, and MPCR were performed on BAL fluid of critically ill patients with pneumonia suspicion. The final diagnosis of infective pneumonia was retained after the medical chart was reviewed by two experts. Pathogen identification rate of direct examination, culture, and MPCR in patients with confirmed pneumonia was compared.

**Results:**

Among the 65 patients with pneumonia suspicion, the diagnosis of pneumonia was finally retained in 53 cases. Twenty nine (55%) were community-acquired pneumonia and 24 (45%) were hospital acquired. Pathogen identification rate with MPCR (66%) was greater than with culture (40%) and direct examination (23%) (*p* =0.01 and *p* <0.001, respectively). When considering only the microorganisms included in the MPCR panel, the pathogen identification rate provided by MPCR reached 82% and was still higher than with culture (35%, *p* <0.001) and direct examination (21%, *p* <0.001). Pathogen identification rate provided by MPCR was not modified in the case of previous antibiotic treatment (66% vs. 64%, NS) and was still better than with culture (23%, *p* <0.001).

**Conclusions:**

The results of this pilot study suggest that in critically ill patients, MPCR performed on BAL fluid could provide higher identification rate of pathogens involved in pneumonia than direct examination and culture, especially in patients having received antimicrobial treatment.

## Background

In critically ill patients with pneumonia, antibiotic therapy is mandatory to improve prognosis and should be administered as soon as possible, In the case of ventilator-associated pneumonia, inappropriate antibiotic therapy might increase the length of stay in intensive care units (ICU) and even double mortality [1]-[3].

The spectrum of antibiotic therapy should be adapted as soon as microbial identification is available. Gram staining of respiratory samples obtained with protected distal sampling, or bronchoalveolar lavage (BAL) is rapid but its sensitivity is low [4],[5]. The results of quantitative culture of these specimens are available only 24 to 48 h later. Furthermore, the sensitivity of culture is decreased by prior antimicrobial therapy, especially if antibiotics have been introduced recently [6],[7].

Recent rapid PCR-based techniques provide information allowing prompt pathogen identification even after initiation of antibiotic therapy. The LightCycler SeptiFast® (Roche Diagnostics, Mannheim, Germany) is a real-time multiplex PCR (MPCR) assay which can identify 20 bacterial and fungal species accounting for up to 95% of cases of bacteraemia (Table 1), from a single whole blood sample. This analysis requires between 5 and 6 h with manual DNA extraction and 4 h with automated extraction [8]. Initially, the LightCycler SeptiFast® was designed to identify rapidly the most important microorganisms responsible for bacteraemia in patients with hematological malignancies [9],[10] or cancer [11], especially during febrile neutropenia [12]. This technique was evaluated using blood samples of patients admitted to the ICU [13], emergency units [14], or ward [15],[16]. These studies have shown that MPCR can be simultaneously used with blood culture-based methods in order to improve pathogen identification rate in patients who had received antimicrobials. A study focusing on patients with infectious endocarditis found that compared to valve culture, MPCR performed on the valve tissue significantly increased pathogen identification [17].

**Table 1:** Bacteria and fungi detected by the multiplex PCR assay

**Gram-positive bacteria**	**Gram-negative bacteria**	**Fungi**
*Staphylococcus aureus*	*Escherichia coli*	*Candida albicans*
*Coagulase*-*negative Staphylococcus*	*Klebsiella* (*pneumoniae*/*oxytoca*)	*Candida tropicalis*
*Streptococcus pneumonia*	*Serratia marcescens*	*Candida parapsilosis*
*Streptococcus* spp.	*Enterobacter* (*cloacae/aerogenes*)	*Candida krusei*
*Enterococcus faecium*	*Proteus mirabilis*	*Candida glabrata*
*Enterococcus faecalis*	*Pseudomonas aeruginosa*	*Aspergillus* (*fumigatus*)
	*Acinetobacter baumannii*	
	*Stenotrophomonas maltophilia*	

The aim of the present study was to determine in ICU patients with pneumonia whether MPCR usually performed on the blood culture could be performed on BAL fluid and could improve pathogen identification rate compared to usual microbiological analysis of BAL fluid.

## Methods

### Setting and patients

We conducted this pilot study in our 15-bed medical ICU. On average thousand patients per year are admitted from the units of the hospital (mainly hepatology with liver transplantation, hematology, oncology, infectious diseases, gastroenterology), emergency unit and mobile emergency unit. For availability issue, we had the kits only during two nonconsecutive periods, from April to September 2009 and from February to July 2010.

This study was approved by the Ethics Committee of the Société de Réanimation de Langue Française (SRLF).

All consecutive patients admitted to the ICU with suspected community-acquired pneumonia (CAP) or hospital-acquired pneumonia (HAP) (including ventilator-acquired pneumonia (VAP)) were eligible. An informed consent was given by the patient or by a legal representative if the patient was unable to consent.

Diagnosis of suspected CAP was based on the International Sepsis Forum Definition of Infection in the ICU [18], using clinical, radiological, and biochemical criteria: cough, dyspnea, chest pain, sputum, fever, tachycardia, tachypnea, confusion, localized dullness, body temperature more than 38.3°C, the presence of persistent crackles during deep inspiration, the presence of pulmonary infiltrate on the hospital admission chest X-ray or appearing within 48 h of hospital stay, and leukocytosis (>10,000/mL) or leukopenia (<4,000/mm^3^).

Suspicion of hospital-acquired pneumonia was based on American Thoracic Society guidelines [19],[20] and was defined as: more than 48 h after hospital admission, new or persistent infiltrate on chest X-ray associated with at least two of the following: purulent tracheal secretions, temperature >38°C, and leukocyte count >11,000 or <4,000/mm^3^.

In the case of clinical and bacteriological suspicion of pneumonia with inconclusive chest X-ray, a chest CT scan was performed to confirm the diagnosis.

### Data collection

For every eligible patient, the following data were systematically recorded: age, gender, simplified acute physiology score II (SAPS II), type of pneumonia (CAP or HAP (including VAP)), underlying immunodeficiency [defined as one of the following: AIDS, cancer or hematological disease with chemotherapy administered less than 30 days before admission, recipient of solid organ (liver) or bone marrow transplant, corticosteroids more than 1 mg/kg/day equivalent prednisone for more than 1 month, and other immunosuppressive therapies], core temperature, septic shock according to international definitions [21], invasive mechanical ventilation, type of pulmonary infiltrate (multilobular or not, alveolar, interstitial or mixed), leukocyte counts, plasma C-reactive protein and procalcitonin levels, cytology of the BAL, administration of a recent antibiotic treatment (defined as a new antibiotic therapy initiated within 48 h before BAL), and results of all microbiological specimens obtained during 2 days before or after BAL and in ICU mortality.

### Procedures and analysis

In our unit, fiberoptic bronchoscopy is performed by a senior intensivist and is therefore available 24 h a day, 7 days a week. Every spontaneously breathing patient with suspected pneumonia underwent fiberoptic bronchoscopy via the nasal route after local anesthesia with topical lidocaine. When the patient was receiving invasive mechanical ventilation, the fiberoptic bronchoscopy was performed through the endotracheal tube under general anesthesia. Bronchoalveolar lavage was performed in the bronchial segment corresponding to the pulmonary infiltrate present on the chest X-ray. In the case of diffuse pulmonary abnormalities, the bronchial segment where the infiltrate was the most important or the middle right bronchus was sampled.

Briefly, five aliquots of 20 mL of 0.9% saline were prepared. The first was discarded as recommended [22]. The others were pooled and separated into three aliquots. The first aliquot was sent to the microbiology lab for direct examination (DE) with Gram coloration and culture (C). The positivity threshold of C after 24 h was 10^4^/mL.

The second aliquot was frozen and kept stored at −20°C for MPCR, and the last one was sent for cytological analysis. Considering that the results provided by MPCR were not taken into account to treat the patient and that the DNA structure remains long-term stable in frozen biological samples [23], MPCR was not performed in real time but LBA samples dedicated to PCR were analyzed six by six using the automated platform.

The dedicated MPCR frozen stored aliquots were thawed, and the tests were performed using 1.5 mL of BAL fluid. The guidelines provided by the manufacturer for blood samples were followed, except that the sample lysis step (of red and white blood cells) was not performed. A positive and a negative control were included in each experiment. Briefly, DNA was extracted using a MPCR Prep Kit (Roche Diagnostics, Mannheim, Germany). Real-time PCR amplification was performed using an MPCR Kit with the LightCycler 2.0 instrument. Internal controls were included in the assay.

As for culture, detection by MPCR of one of the following: *Coagulase-negative Staphylococci* (CNS), *Streptococcus* spp., *Enterococcus faecalis*, *Enterococcus faecium*, and *Candida* spp. was not taken into account unless they were the sole or largely predominant pathogen in immunocompromised patients.

One week after ICU admission, according to the clinical evolution, chest radiograph, and results of DE, C, and other conventional microbiological samples, all medical files were assessed in a blinded manner by two senior physicians to rule in or not the diagnosis of infectious pneumonia. In the case of discrepant conclusions, the data were assessed by a third senior intensivist until a consensus was obtained. Physicians analyzing the data as well as physicians in charge of the patients were blinded to the results of the MPCR.

Based on the clinical history, clinical examination, radiological file, biological results, and of other contemporary microbiological samples (blood cultures, tracheal aspiration, *Streptococcus pneumoniae*, and *Legionella pneumoniae* antigenuria), the diagnosis of infectious pneumonia was ruled in or ruled out and the MPCR results were assessed and compared with the results of DE and C.

### Statistical analysis

Variables are expressed as mean ±1 SD. Proportions were compared using the Fisher exact test for independent samples and the McNemar chi-square test for matched pairs. All statistical tests were two sided at a 5% level of significance. All statistical computations were performed using SAS software version 9.2 (SAS Institute Inc, Cary, NC, USA).

## Results

During the study periods, pneumonia was suspected in 65 patients and finally confirmed in 53 of them (age: 61 ± 16 years, SAPS II: 48 ± 25, invasive mechanical ventilation requirement: 57%, septic shock: 32%, immunosuppression: 45%). The chest X-ray showed the pneumonia in 47 patients. In six patients, the pneumonia was visible only in the chest CT scan. The types of pneumonia broke as follows: CAP (*n* = 29) and HAP (*n* = 24). Among the HAP, nine were VAP (in 2009, it was declared 27 VAP with 16.6% of incidence density and in 2010, 34 VAP with 17.43% of incidence density).

Demographics and clinical characteristics of the 65 patients with suspected pneumonia are summarized in Table 2.

**Table 2:** Characteristics of the patients included in the study

**Pneumonia**	**Infectious (*n*** **= 53)**	**Noninfectious (*n*** **= 12)**
	**CAP = 29**	
	**HAP = 24 (with 9 VAP)**	
**Gender (male to female ratio)**	62.7%	25%
**Mean age (years)**	61.1 ± 16.4	55 ± 15.3
**SAPS II**	48.4 ± 25,1	38.8 ± 17
**Immunodeficiency**	24 (45.3%)	6 (50%)
HIV	8	0
Neoplasia/hematologic disease	6/4	0/2
Systemic disease	4	3
Inflammatory bowel disease	1	1
Liver transplant	1	0
**Immunosuppressive therapy**	14 (26.4%)	8 (66.6%)
Antineoplastic chemotherapy	7	1
Corticosteroids	5	6
Anti-TNF antibodies	0	2
Azathioprine	1	0
**Previous antibiotic therapy**	74%	83%
**Clinical characteristics**		
Temperature <36° or >38°	28 (52.8%)	4 (33.3%)
Confusion	7 (13.2%)	3 (25%)
Shock	18 (33.9%)	4 (33.3%)
Mechanical ventilation	29 (54.7%)	8 (66.6%)
**Radiologic findings**		
Multilobar infiltrates	27 (50.9%)	11 (91.6%)
**Laboratory findings**		
Leucocytes >11,000/mm^3^	31 (58.4%)	11 (91.6%)
Leucocytes <3,000/mm^3^	8 (15%)	2 (15.6%)
CRP	204 ± 139	125 ± 100
PCT >10 ng/mL	7 (13.2%)	3 (25%)
**Histology of BAL**		
Cells per mL	478,941 ± 530 571	202,000 ± 229,242
Polynuclear cells (%)	78.19 ± 11.03	76.81 ± 16.55

CAP, community-acquired pneumonia; HAP, hospital-acquired pneumonia; VAP, ventilator-acquired pneumonia.

Among the 12 patients for whom pneumonia was finally ruled out, alternative diagnosis was: intra-alveolar hemorrhage (*n* = 3), hemodynamic pulmonary edema (*n* = 2), *a vacuo* edema complicating pneumothorax exsufflation (*n* = 1), pulmonary fibrosis (*n* = 1), and acute respiratory distress syndrome or acute lung injury related to extrapulmonary sepsis (*n* = 5). Of these five patients, one had an infection of ascites with septic shock (PCT <0.5 ng/mL), one had a pyelonephritis with septic shock (PCT >10 ng/mL), one had a peritonitis with septic shock (PCT not available), one had a liver abscess without shock (PCT >10 ng/mL), and one had a pyelonephritis without shock (PCT >10 ng/mL).

At the time of the BAL, 39 patients (74%) were receiving recent antibiotic therapy (patients with CAP: 18 (62%), patients with HAP: 21 (87%)). The reasons for this antibiotic treatment were: pneumonia (*n* = 27), bronchitis (*n* = 4), bactaeremia associated with febrile neutropenia (*n* = 2), febrile neutropenia without bactaeremia (*n* = 2), urinary tract infection (*n* = 2), arthritis-associated bactaeremia (*n* = 1), and intra-abdominal infection (*n* = 1).

The results of pathogen identification provided by DE, C, and MPCR are depicted in Tables 3 and 4. Pathogen identification rate provided by MPCR (66%) was significantly higher than the ones provided by DE (23%, *p* <0.001) and C (40%, *p* = 0.01), irrespective of whether pneumonia was community or hospital acquired.

**Table 3:** Comparison of diagnosis provided by direct examination, culture, and MPCR

**Direct examination**	**Culture**	**MPCR**	
+	+	+	*n* = 9
+	+	−	*n* = 1
+	−	+	*n* = 0
+	−	−	*n* = 1
−	+	+	*n* = 6
−	+	−	*n* = 5
−	−	+	n = 20
−	−	−	*n* = 11
Total positive: 12 (23%)	Total positive: 21 (40%)	Total positive: 35 (66%)^$,£^	

53 patients with a final diagnosis of pneumonia. ^$^*p* <0.001 for DE vs. MPCR; ^£^*p* =0.01 for C vs. MPCR.

**Table 4:** Pathogens identified by direct examination, culture, and/or MPCR according to previous administration of antibiotic therapy

**Pt**	**Previous antibiotic therapy**	**Direct exam**	**Culture**	**MPCR**	**Other positive microbiological specimen**	**Final identification**
1	Yes	0	0	*E. coli*/*P. aeruginosa*	TA *E. coli*/*P. aeruginosa*	Yes
					1 day before BAL	
2	Yes	0	0	*S. aureus*	No	Yes
3	No	GPD	*S. pneumoniae* 10^4^/mL	*S. pneumoniae*	No	Yes
4	No	GPD	*S. pneumoniae* 10^5^/mL	*S. pneumoniae*	No	Yes
5	Yes	0	0	*P. aeruginosa*	Blood culture *P. aeruginosa*	Yes
					1 day before BAL	
6	Yes	0	0	*E. cloacae*	No	Yes
7	Yes	0	0	0	AgU *S. pneumoniae*	Yes
					On BAL day	
8	Yes	GNB	*E. coli* 10^3^/mL	*E. coli*	Blood culture *E. coli*	Yes
					On BAL day	
9	Yes	0	0	0	No	No
10	Yes	0	*S. aureus* 10^4^/mL*/P. aeruginosa* 10^3^/mL	*S. aureus/P. aeruginosa*	No	Yes
11	No	0	0	*E. coli*/*Klebsiella* spp./*S. aureus*	TA *S. aureus/Klebsiella* spp.	Yes
					On BAL day	
12	Yes	0	0	*E. cloacae/S. aureus*	TA *E. cloacae*	Yes
					On BAL day	
13	No	GNB	*E. coli* 10^5^/mL	*E. coli*	No	Yes
14	Yes	0	0	0	No	No
15	No	0	*S. pneumoniae* 10^6^/mL	*S. pneumoniae*	No	Yes
16	Yes	0	0	*S. marcescens*/*E. coli*	TA *S. marcescens/E. coli*	Yes
					1 day before BAL	
**17**	**Yes**	***P*.** ***jirovecii***	**0**	**0**	**No**	**Yes**
**18**	**No**	**0**	***Haemophilus*** 10^5^/mL	**0**	**No**	**Yes**
19	No	0	*S. pneumoniae* 10^5^/mL	*S. pneumoniae*	No	Yes
20	Yes	GNB	*E. cloacae* 10^3^/mL	*E. cloacae*	No	Yes
21	Yes	0	0	*S. pneumoniae*	AgU *S. pneumoniae*	Yes
					On BAL day	
22	Yes	0	0	0	No	No
23	No	GPD	*S. pneumoniae* 10^4^/mL	*S. pneumoniae*	Blood culture *S. pneumoniae*	Yes
					On BAL day	
24	Yes	GPD	*S. pneumoniae* 10^3^/mL	*S. pneumoniae*	AgU *S. pneumoniae*	Yes
					1 day after BAL	
25	Yes	0	0	0	No	No
**26**	**No**	**0**	***Haemophilus*** 10^3^/mL	**0**	**No**	**Yes**
27	Yes	0	0	*P. aeruginosa*	Blood culture *P. aeruginosa*	Yes
					On BAL day	
28	Yes	0	0	*E. coli*	No	Yes
29	Yes	0	*S. pneumoniae* 10^2^/mL	*S. pneumoniae*	AgU *S. pneumoniae*	Yes
					The same day	
30	Yes	0	0	*E. coli*	Blood culture *E. coli*	Yes
					1 day before BAL	
**31**	**No**	**0**	***Haemophilus*** 10^5^/mL	**0**	**No**	**Yes**
32	Yes	0	0	*S. aureus*	No	Yes
33	Yes	0	0	*S. pneumoniae*	No	Yes
**34**	**Yes**	**0**	**0**	**0**	**PCR** ***P*.** ***jirovecii***	**Yes**
					4 days after	
35	Yes	0	0	*K. pneumoniae*	No	Yes
36	No	0	*S. aureus* 10^5^/mL	*S. aureus*	No	Yes
37	Yes	0	0	*S. pneumoniae*	No	Yes
**38**	**Yes**	**0**	***H*.*alvei*** 10^4^/mL	**0**	**No**	**Yes**
**39**	**Yes**	**0**	**0**	**0**	**PCR H1N1**	**Yes**
					2 days after	
40	Yes	0	0	*S. aureus*	No	Yes
41	Yes	0	0	*P. aeruginosa*	No	Yes
42	Yes	0	*K. pneumoniae* 10^4^/mL	*K. pneumoniae*	No	Yes
43	No	0	0	*S. pneumoniae*	AgU *S. pneumoniae*	Yes
					The same day	
44	Yes	0	0	0	No	No
**45**	**Yes**	**0**	***L. pneumophila***	**0**	**No**	**Yes**
**46**	**Yes**	***P*.** ***jirovecii***	**0**	**0**	**No**	**Yes**
47	Yes	GPC	*S. epidermidis* 10^5^/mL	*S. epidermidis*	Blood culture *S. epidermidis*	Yes
					1 day before BAL	
48	No	GNB	*E. coli* 10^6^/mL	*E. coli*	No	Yes
**49**	**No**	**GNB**	***Haemophilus*** 10^5^/mL	**0**	**No**	**Yes**
50	Yes	0	0	*S. marcescens*	TA *S. marcescens*	Yes
					On BAL day	
51	Yes	0	0	0	TA ***Haemophilus***	Yes
					1 day before BAL	
52	Yes	0	0	0	TA *S. pneumoniae*	Yes
					On BAL day	
53	Yes	0	0	*E. coli*	Blood culture *E. coli*	Yes
					1 day after	

GPD, gram-positive diplococci; GNB, gram-negative bacilli; GPC, gram-positive cocci; TA, tracheal aspirate; AgU *S. pneumoniae*, urinary antigen test for *S. pneumoniae*. The date following the samples corresponds to the day when the sample was performed. Bold lines represent the pathogens not included in the detection panel of the MPCR assay.

When DE was negative, MPCR was markedly more often positive than C (63% vs. 27%, *p* = 0.0007). Using data provided by other microbiological tests (*Pneumocystis jirovecii PCR*, H1N1 PCR, tracheal aspirate during the 48 h preceding BAL, urinary pneumococcal antigen), a pathogen identification was obtained in five supplemental cases, resulting in a final pathogen identification in 48/53 cases (90%). All the five patients, for whom all microbiological examinations were negative, were receiving recent antibiotic therapy when BAL was performed.

Considering the 20 cases (38%) of pneumonia for which pathogen identification was provided by MPCR but neither by DE nor by C, 25 bacterial species were identified (Table 3): *S. pneumoniae* (*n* = 4), *Escherichia coli* (*n* = 6), *Staphylococcus aureus* (*n* = 5), *Pseudomonas aeruginosa* (*n* = 4), *Serratia* (*n* = 2), *Klebsiella* (*n* = 2), and *Enterobacter cloacae* (*n* = 2). In these 20 patients, MPCR results were confirmed by other microbiological investigations for 11 of them [blood culture (*n* = 4), previous tracheal aspirate during the 48 h preceding BAL (*n* = 5), urinary pneumococcal antigen (*n* = 2)]. Nine of these 11 patients were receiving recent antibiotic therapy when BAL was performed. The remaining nine patients, for whom MPCR was the only positive microbiological test, were all receiving recent antibiotic therapy when BAL was performed.

Among the 48 patients with a microbiological identification provided by at least one method, MPCR did not provide any pathogen identification in 13 of them. All the pathogens retained as causing these 13 pneumonia were not included in the panel of strains detected by MPCR except *S. pneumoniae* (in two patients). Pathogens not identified by MPCR were identified by 1) direct examination or culture: *P. jirovecii* (*n* = 2), *Legionella pneumophila* (*n* = 1), *Haemophilus influenzae* (*n* = 4), and *Hafnia alvei (n =* 1); 2) tracheal aspirate obtained 24 h earlier (*S. pneumoniae*(*n* = 1), *H. influenzae* (*n* = 1); 3) specific PCR *H1N1* virus (*n* = 1), PCR *P. jirovecii* (*n* = 1); or 4) urinary pneumococcal antigen (*n* = 1). When restricting the analysis to the microorganisms theoretically detected by MPCR, pathogen identification rate provided increased to 82%, while it was 35% (*p* <0.001) and 21% (*p* <0.001) for culture and direct examination, respectively.

In patients for whom pneumonia diagnosis was finally ruled out, MPCR detected a pathogen in 5/12 patients (42%). This was considered as colonization.

**Figure 1:**
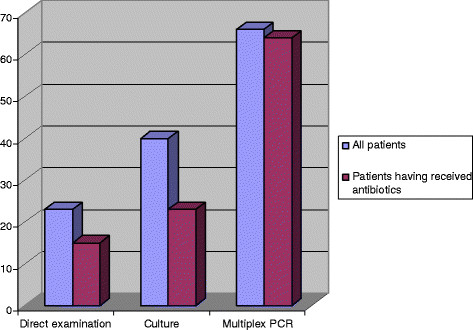
**Identification rate (percentage) of pathogen provided by direct examination, culture, and multiplex PCR.** In all patients and in patients having received previous administration of antibiotics.

When patients had received recent antibiotic therapy prior to BAL, pathogen identification rate with MPCR was not modified (64%) and remained higher than with direct examination (15%, *p* <0.001) or culture (23%, *p* <0.001) (Figure 1).

## Discussion

The present study is the first to assess whether MPCR performed on BAL fluid enables pathogen identification complementary with the direct examination and the culture in patients with severe pneumonia. Recently published guidelines recommend antibiotic administration within 1 h for patients suspected of having septic shock or severe sepsis [24]. During severe infection, empirical antibiotic therapy is inappropriate in roughly one-third of cases, and this substantially increases mortality and hospital length of stay [25]. In the setting of infection, any tool enabling prompt and accurate documentation of pathogen might theoretically reduce mortality and may serve to improve hospital resource use [25].

During two nonconsecutive periods, in unselected critically ill patients with community or hospital-acquired pneumonia, we performed, as usually done in our unit, bronchoalveolar lavage during fibroscopic examination. Results of direct examination using conventional staining and culture were provided as soon as possible and guided antibiotic therapy. A MPCR processed on BAL fluid was performed after a delay of several days, and its results were not taken into account to modify antibiotic therapy management.

Final diagnosis of pneumonia relied on *a posteriori* analysis of clinical, radiological, and microbiological data. This analysis was conducted by two senior physicians who were blinded to MPCR results.

The present data also suggest that MPCR can be done on samples having been kept frozen for several days. This study increases the number of biological fluid on which MPCR can be of interest. Reliable results obtained more quickly with MPCR (performed on urine samples) than with culture have been observed during urinary tract infection, [26]. Similar date was observed on other fluids (biliary, pleural, peritoneal, or intra-articular fluid) [27],[28].

Multiplex PCR significantly increased the identification rate of pathogen causing pneumonia, compared with usual culture alone. Increased pathogen identification rate provided by MPCR compared to conventional cultures has been reported during the course of bloodstream infections [9],[16],[29],[30], febrile neutropenia [12], and persistent fever [31].

In the present study, pathogen identification rate would have probably be higher if the panel of the MPCR had included microorganisms frequently causing pneumonia like *H. influenzae*, *L. pneumophila*, *H. alvei*, *respiratory viruses*, and *P. jirovecii*. These pathogens were considered as the cause of pneumonia in 8/18 of the cases for which MPCR did not provide microbiological identification, while direct examination and/or culture did.

The contribution of MPCR to pathogen identification was of particular interest in patients who had received recent antibiotic treatment. Pathogen identification rate provided by MPCR was not modified (compared to identification rate observed in all patients) while it decreased for direct examination and culture. This issue is in keeping with which was observed in other types of infection [32]-[34].

Owing to its ability to provide pathogen identification more frequently than direct examination and culture MPCR could allow earlier initiation of appropriate antibiotic therapy [29].

In our study, if the MPCR has been done in real time, the initial antibiotic therapy, appropriate in 87% (46/53), would have been changed in nine patients: deescalation in seven cases (anti *staphylococcus* antibiotic therapy (*n* = 2), anti *P. aeruginosa* antibiotic therapy (*n* = 1), anti *S. pneumoniae* antibiotic therapy (*n* = 4), change of antibiotic therapy in two cases (anti *staphylococcus* antibiotic)).

Direct examination provides information in less than 2 h, but the diagnostic input of direct examination performed on BAL fluid was weak in our study, and it identified pathogens in only 23% of patients.

Our study has nevertheless several limitations. First, this was a monocenter study performed on a limited number of nonconsecutive patients. These promising data need to be confirmed on more large ICU population, especially in the subgroup of patients (immunosuppressed patients). Second, due to logistical and economical consideration, MPCR analysis was pooled and was not performed in real time. A trial assessing the impact of MPCR performed in real time in critically ill patients with pneumonia warrants consideration. Third, the time required by the technique (4 to 6 h) to provide microbiological results could be considered excessive, since currently available fully automated PCR platforms provide results in 1 h. It should be highlighted that these latter PCR kits use a single probe and can only identify a single microorganism (*S. aureus*, *Mycobacterium tuberculosis*, or *Clostridium difficile*[35]-[38]). On the other hand, mass spectrometry yields results in a very short time (few minutes), but it requires that the microorganism has been isolated [39],[40].

Multiplex PCR has nevertheless some limits. First, MPCR performed on BAL fluid cannot give quantitative results and is therefore unable to differentiate colonization from infection.

In considering the number of cycles required for obtaining positivity, it could be possible to approach the distinction between colonization and infection (similarly to what is done for *P. jirovecii*). However, in patients having received antibiotic therapy prior to BAL but having suggestive anamnesis, clinical examination, evolution radiological and biological results, and of other contemporary microbiological results, should the diagnosis of pneumonia be ruled out when culture provided less colony forming unit than the threshold?

Culture remains the gold standard of routine investigation to obtain quantitative data and confirm infection, especially in the case of ventilator-acquired pneumonia [41].

However, in association with C, MPCR appears highly relevant to identify organisms likely involved in pneumonia, allowing thus administration of the most appropriate antibiotic therapy.

On other hand, unlike the culture, MPCR does not provide antibiotic susceptibility testing and culture remains fundamental.

Cost must also be taken into consideration, since MPCR is three times more expensive than C, and the cost-effectiveness of a diagnostic strategy using MPCR has to be assessed. It may be appropriate to limit MPCR to the most severely ill patients in the ICU [42]. On the other hand, MPCR which could decrease the use of antibiotics [43] might decrease the rate of inappropriate antibiotic therapy and therefore decrease the antimicrobial resistance development [25].

## Conclusions

In conclusion, our study conducted in patients with severe pneumonia is the first to assess whether MPCR performed on BAL fluid enables pathogen identification. Our data suggest that MPCR provides higher identification rate than conventional microbiological methods. MPCR could be particularly valuable in patients with recent antibiotic treatment.

## Competing interests

The authors declare that they have no competing interests.

## Authors’ contributions

JLB, JT, RD, and EM designed the study. JLB, JT, RD, AG, HAO, BG, GO, and EM collected the data. JLB wrote the draft of manuscript. FC performed the statistical analysis. All the authors corrected and approved the manuscript.
